# Isolation of *Waddlia malaysiensis*, A Novel Intracellular Bacterium, from Fruit Bat (*Eonycteris spelaea*)

**DOI:** 10.3201/eid1102.040746

**Published:** 2005-02

**Authors:** Paul K.B. Chua, John E. Corkill, Poh Sim Hooi, Soo Choon Cheng, Craig Winstanley, C. Anthony Hart

**Affiliations:** * University of Liverpool, Liverpool, United Kingdom;; † National Public Health Laboratory, Kuala Lumpur, Malaysia;; ‡ University of Malaya, Kuala Lumpur, Malaysia

**Keywords:** antimicrobial susceptibility, Chlamydia, Waddlia, fruit bat, 16S rDNA inclusions, research

## Abstract

An obligate intracellular bacterium was isolated from urine samples from 7 (3.5%) of 202 fruit bats (*Eonycteris spelaea*) in peninsular Malaysia. The bacterium produced large membrane-bound inclusions in human, simian, and rodent cell lines, including epithelial, fibroblastlike, and lymphoid cells. Thin-section electron microscopy showed reticulate bodies dividing by binary fission and elementary bodies in the inclusions; mitochondria surrounded the inclusions. The inclusions were positive for periodic acid-Schiff stain but could not be stained by fluorescein-labeled anti–*Chlamydia trachomatis* major outer membrane protein monoclonal antibody. The bacterium was resistant to penicillin and streptomycin (MICs >256 mg/L) but susceptible to tetracycline (MIC = 0.25 mg/L) and chloramphenicol (MIC = 0.5 mg/L). Sequence analysis of the 16SrRNA gene indicated that it was most closely related to 2 isolates of *Waddlia chondrophila* (94% and 96% identity). The 16S and 23S rRNA gene signatures were only 91% identical. We propose this novel bacterium be called *W. malaysiensis*.

## Abstract 2

A novel obligate intracellular bacterium was isolated from urine samples from fruit bats *(Eonycterisspelaea)* in peninsular Malaysia.

An estimated 1,415 microbes are infectious for humans (*1*). Of these, 868 (61%), are considered to be zoonotic; overall, zoonotic pathogens are twice as likely to be associated with emerging diseases (*1*). Wildlife have been increasingly recognized as important reservoirs of potentially zoonotic microorganisms (*2**,**3*). In particular, bats have been shown to be both important reservoirs and vectors of pathogens. These pathogens include viruses such as rabies (*4*), European lyssavirus (*5*), Hendra (*6*) and Menangle (*7*) viruses in Australia, Nipah and Tioman viruses in Malaysia (*8**,**9*), hantaviruses in Korea (*10*), a number of different bunyaviruses, flaviviruses, and alphaviruses. Moreover, solitary microchiropteran bats are prime contenders as reservoirs of Marburg and Ebola viruses. In addition, bats have been identified as reservoirs of fungi such as *Histoplasma capsulatum* and *Coccidioides immitis*. However, apart from leptospirosis (*11*) and some studies on enteric flora and pathogens (*12**–**14*), little is known of the bacteria that infect and are excreted by bats.

As part of an investigation into the reservoir of Nipah virus in Malaysia (*8**,**9**,**15*), a novel chlamydialike bacterium was isolated from the urine of *Eonycteris spelaea*; the Lesser Dawn Bat (*16*). This bat is a generalist nectivore that travels tens of kilometers from its cave-roosting sites to feed (*16*). It is found throughout Burma, Indonchina, the Philippines, Malaysia, Indonesia, Nepal, and northern India. Little is known of the potential pathogens harbored by *E. spelaea*, but 1 survey of lyssavirus infection of bats in the Philippines did not detect virus in brain sections or neutralizing antibody to rabies or Australian bat lyssavirus in serum from *E. spelaea* (*17*). Neither Nipah nor Tioman viruses have been isolated from *E. spelaea*, and detecting this chlamydialike bacterium was a chance finding (*15*). We describe the isolation and characterization of this novel bacterium and propose that it be given the name *Waddlia malaysiensis* since it was first isolated in Malaysia.

## Material and Methods

### Collection of Samples and Isolation of the Bacterium

As part of an investigation into the reservoir of Nipah virus (*8**,**9*), we made 3 field trips from May to July 1999 to a colony of fruit bats (*E. spelaea*) roosting in a cave (Gua Tempurong) situated 25 km from the initial Nipah outbreak in Perak, northern peninsular Malaysia. The first visit was to observe the fruit bats' roosting behavior, in particular, timing of return to roost, leaving for feeding, and urination and defecation habits. In the second and third visits, clean plastic sheets (1.5 × 3 m) were suspended over areas where the bats had been observed previously to urinate and defecate (*15*). The sheets were suspended ≈0.5 m above the ground and held taut with 4 metal rods. The sheets and rods were put in place 30 min before the bats were expected to return to roost. Sterile cotton swabs were used to collect the urine as soon as it fell onto the plastic sheets. The swabs were then placed into virus transport medium (2 mL: ICN Biomedicals Inc, Irvine, CA, USA), containing 1% bovine albumin hydrolysate, amphotericin B (20 µg/mL), penicillin G (100 U/mL), and streptomycin (50 µg/mL). The samples were transported at 4°C to the laboratory on the day of collection. Each swab, in transport medium, was gently vortexed, and 200 µL of the medium was transferred into individual wells of a 24-well tissue culture plate (Sterilin, Stone, U.K.) preseeded with 1 × 10^5^ Vero cells in Eagle's minimal essential medium (Sigma, Basingtoke, U.K.). The plates were sealed and incubated at 37°C. The culture was examined daily for cytopathic effect (CPE) with phase-contrast microscopy. Isolates were stored at –70°C, and 1 strain was chosen at random for further characterization and transported to Liverpool at –20°C.

### Microbiologic Characteristics

To determine the range of cells susceptible to infection, different cells were cultured in 25-m^2^ plastic flasks (Becton Dickinson, Basingstoke, U.K.) in 199 medium (Sigma) with 2% (vol/vol) fetal calf serum but no added antimicrobial agents. Because the bacterium replicated so rapidly, including chlorhexidine, normally used in culture of *Chlamydia trachomatis* to prevent overgrowth of Vero cells, was not necessary. Approximately 10^7^ bacterial cells (as determined by electron microscopic count) were added to each flask of cells and incubated at 37°C in air with 5% CO_2_ and examined daily for CPE. For each cell line, growth was determined by both phase-contrast microscopy and demonstration of inclusions by thin-section electron microscopy. A variety of human (Hep-2, HEK, MRC-5, A549 and an Epstein Barr virus (EBV)–transformed human B-lymphoblastoid line), simian (Vero, LLC-MK2), and rodent (3T3, BHK) cell lines were used. Attempts were also made to grow the bacteria on 7% horse blood Columbia agar plates in air with 5% CO_2_ and anaerobically at 37°C for 72 h.

To determine antimicrobial susceptibility, coverslip cultures of Vero cells were prepared as described previously except that chlorhexidine was omitted from the growth medium (*18**,**19*). After 48 h of incubation, the growth medium was removed and ≈10^5^ bacteria (in 0.5 mL medium) were added to each vial containing the coverslip monolayer of Vero cells. After absorption (without centrifugation) for 30 min, fresh 199 medium with 2% fetal calf serum, which incorporated doubling dilutions of antimicrobial agents from 1 mg/L down to 0.06 mg/L and doubling increases in concentration from 1 mg/L to 256 mg/L, was added. The antimicrobial agents used were chloramphenicol, tetracycline, penicillin G, and streptomycin. The coverslip cultures were incubated at 37°C for 72 h; they were then methanol-fixed and Giemsa-stained as described previously (*19*). The MIC of an antimicrobial agent was defined as the lowest concentration required to inhibit the formation of inclusions.

To determine staining characteristics, coverslip cultures of Vero cells were infected with ≈10^5^ bacteria. After 48 h of culture, the cells were methanol-fixed and stained by Giemsa, periodic acid-Schiff (PAS), or immunofluorescence staining by using a monoclonal antibody directed against the major outer membrane protein of *C. trachomatis* (Microtrak, Trinity Biotech, Bray, Ireland) as described previously (*18**,**19*). For thin-section electron microscopy, infected cells were fixed in cacodylate-buffered glutaraldehyde (2%), scraped from the flask, postfixed through increasing concentrations of ethanol (to 100% vol/vol), and then araldite embedded. Thin-sections were stained in uranyl acetate and Reynold's lead citrate and examined with a Philips 301 electron microscope. For negative-stain electron microscopy, suspensions were placed on a Formvar-coated grid and stained in phosphotungstic acid.

### Genomic Characteristics

Total DNA was extracted from a 72-h culture of the bacterium in Vero cells. The infected cells were scraped from a 25-cm^2^ tissue culture flask (Becton Dickinson, Basingstoke, U.K.) into 2 mL 199 medium without fetal calf serum. One milliliter of this mixture was centrifuged at 13,000 × *g* for 30 min, and the pellet was suspended in 250 µL of 5% wt/vol Chelex-100 resin slurry (BioRad, Hemel Hempstead, U.K.). This suspension was boiled for 15 min, followed by centrifugation at 13,000 ´ *g* for 10 min; the supernatant was then removed and stored at –20°C until used.

For analysis of the 16S rRNA gene, a 1,526-bp amplicon was produced by using primers 16S-FOR and 16S-REV (Table 1) as described by Rurangirwa et al. (*20*). The amplicon was excised from the agarose gel and purified by using a gel purification kit (Qiagen, West Sussex, U.K.). The amplicon was cloned into a cloning vector, pGEM-T (Promega, Southampton, U.K.) and transformed into *Escherichia coli*. Full-length sequencing of the 1,526-bp amplicon within the cloning vector was achieved by using overlapping internal primers (F1-F4 forward and R1-R4 reverse, Table 1). 16S rRNA signature sequence, 16S-23S rRNA intergenic space, and 23S rRNA domain I signature sequence polymerase chain reaction (PCR) were carried out by using the method of Everett et al. (*21*) with the primers shown in Table 1. In each case, PCR amplification was performed in 50-µL volumes. All primers were added at 20 pmol per assay; PCR buffer (plus 1.5 mmol/L MgCl_2_), Q solution, and Taq polymerase were obtained from Qiagen Ltd (Crawley, U.K.). The presence of the *murA* protein signature was sought by PCR by using primer *murA*-for and *murA*-rev (Table 1), which amplifies a 690-bp fragment of the UDP-N-acetylglucosamine 1-carboxyvinyltransferase gene of *Waddlia chondrophila* (*22*). In this case, PCR was attempted by using a range of Mg^2+^ concentrations from 1.5 to 4.0 mmol/L. Primers to amplify a 331-bp segment of the *sctN* gene were designed by alignment of the *sctN* genes of *C. trachomatis* (AE001337), *C. pneumoniae* (AE002167), and *C. muridarum* (AE002271). The *sctN* gene encodes a type III secretion system ATPase, which is highly conserved among these bacteria (*23*). Sequence determination was performed by using an automated DNA sequencer (ABI PRISM 377; Perkin-Elmer, Warrington, U.K.) and was analyzed by using commercial software (Lasergene: DNAStar Inc., Madison, WI, USA).

**Table 1:** Oligonucleotide primers for PCR and sequencing*

Gene target	Primer sequence
PCR	
16S rRNA (1,526 bp from ref. 20)	
16S-FOR	5´ AGA GTT TGA TCC TGG 3´
16S-REV	5´ TAC CTT GTT ACG ACT T 3´
Tm = 55°C	
16S rRNA signature sequence (298 bp from ref. 21)	
16S1GF	5´ CGG CGT GGA TGA GGC AT 3´
16S1GR	5´ TCA GTC CCA GTG TTG GC 3´
Tm = 51°C	
16S – 23S rRNA signature sequence (1 kbp from ref. 21)	
16SF2	5´ CCG CCC GTC ACA TCA TGG 3´
23S1GR	5´ TGG CTC ATC ATG CAA AAG GCA 3´
Tm = 61°C	
23S rRNA signature sequence (627 bp: domain I from ref. 21)	
23S1GR	5´ TGG CTC ATC ATG CAA AAG GCA 3´
Tm = 61°C	
*MurA* signature sequence (690 bp from ref. 22)	
*murA*-for	5´ GTN GGN GCN ACN GAR AA 3'
*murA*-rev	5´ GCC ATN ACR TAN GCR AAN CCN GC 3´
Tm = 55°C	
*sctN* (331 bp)	
*sctN* FOR	5' AGA RGG AAT GAA ACG TTC 3'
*sctN* REV	5' GGC TCR TTC ATA TCA TC 3'
Tm = 58°C	
Sequencing	
16S *rRNA* (1,526 bp)	
Forward:	
F1 (M13)	5 GTT TTC CCA GTC ACG ACG TTG TA 3´
F2	5´ GCT CAC CAA GGC TAA GAC GTC 3´ (277-298)
F3	5´ CTA GCT TTG ACC TGA CGC TGA T 3´ (752-774)
F4	5´ GAA TCT GCA ACT CGG CTC CAT G 3´ (1323-1345)
Reverse:	
R1 (M13)	5´ TTG TGA GCG GAT AAC AAT TTC 3´
R2	5´ CAT CCT AAA TGC TGG CAA C 3' (392-373)
R3	5´ CAC CGC TAC ATG TGG AAT TCC 3´ (843-822)
R4	5´ GAT CCT CTC TAG CAC CAT ATC C 3´ (1358-1336)

*PCR, polymerase chain reaction.

For phylogenetic analyses, sequence data on complete 16S rRNA genes for each of the Chlamydiales genera were retrieved from GenBank and aligned with ClustalW (*24*). The phylogenetic tree was generated from the alignment by using the genetic distance-based neighbor-joining algorithms of the Data Analysis in Molecular Biology software (DAMBE; http://web.hku.hk/~xxia/software/software.htm). Sequence input order was randomized, and 100 datasets were examined by bootstrapping resampling statistics.

## Results

During the second and third field visits to Gua Tempurong, 206 urine samples were obtained (93 in the second and 113 in the third field visit) from individual bats. A total of 7 urine samples (all from the third visit) produced a characteristic CPE on Vero cells after 5 to 7 days of culture. The same CPE was identified for each of the 7 isolates. One (G817) was therefore selected at random for further characterization.

**Figure 1:**
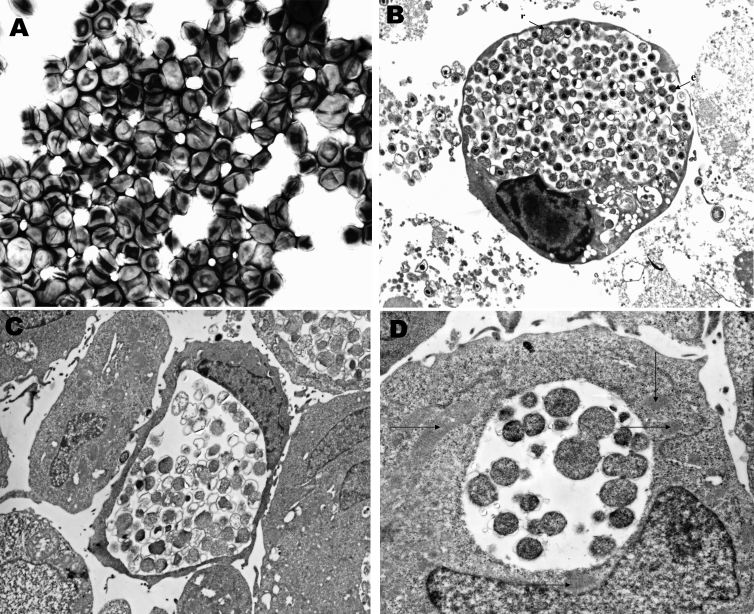
A, Negative stain electronmicrograph of *Waddlia malaysiensis* elementary bodies. B–D, Thin-section electronmicrographs of cells infected with *W. malaysiensis*. B, large inclusion with elementary(e) and reticulate(r) bodies in HEK cells 72 h postinfection. C, a large inclusion in Epstein Barr virus-transformed human B-lymphocytes. D, dividing reticulate bodies in HEK cells 48 h postinfection in an inclusion with numerous surrounding mitochondria (arrow).

On negative-stain electron microscopy of the supernatant from G817 cultured on Vero cells, small bacterial cells (0.4–0.6 µm) resembling chlamydial elementary bodies were seen (Figure 1A). Inclusions visible by phase-contrast microscopy could be detected within 48 to 72 h postinfection of Vero cells. Similar inclusions could be seen after infection of human lung (MRC-5, A549), kidney (human embryo kidney [HEK]), laryngeal (HEp-2), and B-lymphoblastoid cells lines; and of simian kidney (LLC-MK2) and rodent epithelial (3T3, BHK) cell lines. Figures 1A and 1B show large inclusions in HEK- and the EBV-transformed human B-lymphoblastoid cell lines, respectively. In Figure 1B, mixtures of reticulate and elementary bodies are visible. A thin-section electron micrograph of an earlier stage of infection of HEK cells (48 h postinfection, Figure 1D) shows a collection of reticulate bodies with evidence of replication by binary fission. Mitochondria can be seen in close proximity. The bacterium could not be cultured on blood or chocolate agar, aerobically or anaerobically, when incubated for up to 7 days, nor did it have catalase or oxidase activities.

Inclusions could be stained by both Giemsa and PAS but not by the Mikrotrak immunofluorescence system, which recognizes the *C. trachomatis* major outer membrane protein. MICs of tetracycline and chloramphenicol were 0.25 mg/L and 0.5 mg/L, respectively, but streptomycin (256 mg/L) and penicillin G (256 mg/L) did not inhibit the formation of inclusions at therapeutically achievable levels.

**Figure 2:**
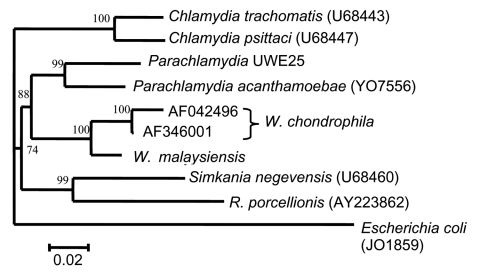
Phylogenetic relationships of *Waddlia malaysiensis* to other Chlamydiales.

All of the 16S rRNA gene, the 16S-23S rRNA intergenic spacer region, and the 627-bp domain I of the 23S rRNA gene were sequenced in both directions. This sequence of 2379 bp has been lodged in GenBank with the accession number AY184804. A BLAST search indicated that a 1,552-bp sequence of the bacterium's 16S rRNA gene had 96% and 94% identity with two 16S rRNA sequences from *W. chondrophila* (AF 346001 and AF 042496). The 16S rRNA (298-bp) and 23S rRNA (627-bp) gene signatures had 91% identity with the 2 *W. chondrophila* sequences deposited in GenBank. The 16S-23S rRNA intergenic space of the bat isolate was 223 bp compared to 213 bp (AF042496) and 217 bp (AF346001) for *W. chondrophila*. Figure 2 shows a neighbor-joining dendogram demonstrating the relationships of the novel bat bacterial isolate to other members the Chlamydiales. This indicates that the novel bacterium is most closely related to, but distinct from, *W. chondrophila*. No PCR amplicons were detected on amplification of either *murA* or *sctN*. When DNA from *C. trachomatis* (lymphogranuloma venereum strain L1) was used as positive control, amplicons of the correct size were detected.

## Discussion

Members of the order Chlamydiales are obligate intracellular bacteria. Recently, a suggestion to revise and update their classification has been made (*21*). This revision was based on comparisons of 16S rRNA and 23S rRNA genes, and it split the Chalmydiales into 4 families, *Chlamydiaceae*, *Simkaniaceae*, *Parachlamydiaceae*, and a family now named *Waddliaceae* (*20*), which has *W. chondrophila* as the prime member (Table 2). This scheme of nomenclature has largely been accepted, although splitting the family *Chlamydiaceae* into 2 genera, *Chlamydia* and *Chlamydophila*, raised some concerns (*25*). The Chlamydiales are an expanding group of bacteria with new genera and species increasingly being described and detected in a wide array of hosts (*26**,**27*). Recent examples include *Rhabdochlamydia porcellionis*, isolated from terrestrial isopods, which is related to but not entirely within the family *Simkaniaceae* (*28*), and 2 insect-associated chlamydia, *Fritschea bemesia* and *F. eriococci* in the family *Simkaniaceae* (*29*). In addition, a number of Chlamydiales endosymbionts have been recovered from human clinical and environmental isolates of *Acanthamoeba* spp. that are related to the *Parachlamydiaceae* (*30*). Indeed for one of these, UWE25, the full genome has been sequenced (*31*). Analyses of 16S rRNA, 23S rRNA genes, and the 16S-23S intergenic space indicate that the bacterium we have isolated from fruit bats is most closely related to the *Waddliaceae* (Figure 2). There are, however, some similarities and differences between our isolate and *W. chondrophila*. *W. chrondrophila* has been isolated twice from cattle, and the bacteria were obtained from a first-trimester bovine abortion in the United States (*20*) and a septic stillborn calf in Germany (*32*). The bacterium from the United States was isolated initially by culture on bovine turbinate cells (*20*), but the German isolate was able to grow in human diploid fibroblasts, simian (Buffalo Green Monkey, and murine [McCoy]) cells lines (*32*). Our bat isolate was able to grow in a wide range of cell types from different anatomic sites and animal species. Some evidence suggests that *W. chondrophila* also has a wide host cell range, but not all possibilities have been tested. There is also recent evidence, based on 16S rDNA amplification, of *W. chondrophila* in an Australian mammal, Gilbert's Potoroo (*33*). Like *W. chondrophila*, our isolate was resistant to penicillin G and streptomycin (MICs >256 mg/L) and could not be stained by immunofluorescence using monoclonal anti–*C. trachomatis* antibodies. However, in contrast to 1 report (*20*), the bat bacterial inclusions stained intensely with PAS stain. This stain reacts with the glycogen matrix elaborated by *Chlamydiaceae* when growing intracellularly. The bat isolate is sensitive to tetracycline (MIC 0.25 mg/L) and chloramphenicol (MIC 0.5 mg/L) at concentrations that are clinically achievable and similar to those needed to cure infections by *C. trachomatis*. No evidence for the presence of one of the key genes (*sctT*) of the pathogenicity island-associated type III secretion system of *C. trachomatis* was found in *W. malaysiensis*; however, this does not mean that no such island is present. Three genes (*sctT*, *sctN*, and *sctV*) from a type III secretion system have been described in the *Parachlamydia*-like endosymbiont UWE25, and sufficient differences exist in the nucleotide and putative amino acid sequences of these, when compared to those of *C. trachomatis* (*31*), that our primers would not amplify it.

**Table 2:** Current status of the Chlamydiales

Family	Genus and species	Biovars	Host/animal disease*
I. *Chlamydiacae*	Chlamydia trachomatis	Serovars A–K	Humans: trachoma, STI
		Serovars L_1_–L_3_	Humans: STI
	C. muridarum	–	Mice: proliferative ileitis
	C. suis	–	Swine: conjunctivitis: pneumonia
	Chlamydophila psittaci	Serovars A–H	Birds, cattle: pneumonia†
	C. pneumoniae	3 biovars	Humans, koala, equines: pneumonia, conjunctivitis
	C. pecorum		Wide host range and disease manifestation
	C. felis	? 2 biovars	Cats: rhinitis†
	C. caviae		Guinea pigs: conjunctivitis
	C. abortus		Sheep, cattle, goats: abortion†
II. *Parachlamydiacieae*	Parachlamydia acanthamoebae		Amoebae: RTI
	Neochlamydia hartmannellae		Amoebae
	Numerous others including UWE25		
III. *Waddliaceae*	Waddlia chondrophila		Cattle, potoroos: abortion
IV. *Simkaniae*	Simkania negevensis		Amoebae, humans: RTI
	"*Candidatus Fritschea bemesiae*"		Whitefly
	"*Candidatus F. eriococci*"		Whitefly
V. *Chlamydialike organisms*	"*Candidatus Rhabdochlamydia porcellionis*"		

*STI, sexually transmitted infection; RTI, respiratory tract infection; –perhaps (i.e., disputed); ?, may be 2 biovars but not confirmed. †Indicates zoonotic potential.

Negative-stain electron microscopic examination of the bat bacterium released from Vero cells showed small cocci indistinguishable from the elementary bodies of *C. trachomatis*. On thin-section electron microscopy of infected cells, large numbers of intracellular bacteria could be seen within membrane-bound inclusions. In mature inclusions in all cell types tested, mixtures of elementary and reticulate bodies were found. In less mature inclusions, dividing reticulate bodies were present, and mitochondria could be seen around the inclusion (Figure 1D). The species name of *W. chondrophila* was derived from the collections of mitochondria around the intracellular inclusions. The bat isolate was closest to the 2 *W. chondrophila* isolates made from cattle on the basis of 16S rRNA gene comparisons (96% and 94% identity). The 16S rDNA and 23S rDNA gene signature sequences also placed the bat bacterium close, to but not identical to, *W. chondrophila* (91%); in addition, the 16S – 23S rRNA intergenic space was slightly longer than for *W. chondrophila*. Thus, the bat isolate is part of the genus *Waddlia*, and we propose the name *Waddlia malaysiensis* for it since it was first detected in Malaysia. The organism appears sufficiently distinct from *W. chondrophila* to justify a different species assignment. It is PAS positive, does not have the *mur*A signature of *W. chondrophila*, and has differences in the 16S – 23S rRNA genomic regions. The collection of mitochondria in proximity to inclusions that gave *W. chondrophila* its species name was also exhibited by *W. malaysiensis* and might therefore be a characteristic of the genus *Waddlia*.

The Chlamydiales infect a wide range of animals including humans (*27**,**34*). Some pathogens such as *C. trachomatis* appear to solely affect humans; others affect only animals; and a sizeable number are zoonotic pathogens (Table 2). *W. chondrophila* has been isolated from aborted cattle fetuses in the United States and German (*20**,**32*) but has also been detected in an apparently healthy Potoroo in Australia (*33*). Recent serologic evidence has suggested a strong statistical association between high titers of *W. chondrophila* antibodies and bovine abortion (*35*). Members of the genera *Parachlamydia* and *Simkania* infect protozoa such as amoebae and can cause disease in humans (*30**,**36**,**37*). In this respect, evidence exists for replication of *W. chondrophila* in amoebae (*38*), which suggests that it might fall into the group of environmentally preadapted pathogens, as has been suggested for *S. negevensis* (*39*) and *C. pneumoniae* (*40*). Whether *W. malaysiensis* can grow in amoebae and has zoonotic potential remains to be determined.

## References

[1] Taylor LH, Latham SM, Woolhouse ME. Risk factors for human disease emergence. Philos Trans R Soc Lond B Biol Sci. 2001;356:983–9. 10.1098/rstb.2001.0888 11516376 PMC1088493

[2] Frohlich K, Thiede S, Kozikowski T, Jakob W. A review of mutual transmission of important infectious diseases between livestock and wildlife in Europe. Ann N Y Acad Sci. 2002;969:4–13. 10.1111/j.1749-6632.2002.tb04343.x 12381556

[3] Simpson VR. Wild animals as reservoirs of infectious diseases in the UK. Vet J. 2002;163:128–46. 10.1053/tvjl.2001.0662 12093188

[4] Paez A, Nunez C, Garcia C, Boshell J. Molecular epidemiology of rabies enzootics in Colombia: evidence for human and dog rabies associated with bats. J Gen Virol. 2003;84:795–802. 10.1099/vir.0.18899-0 12655080

[5] Fooks AR, Finnegan C, Johnson N, Mansfield K, McElhinney L, Manser P. Human case of EL type 2 following exposure to bats in Angus, Scotland. Vet Rec. 2002;151:679. 12498413

[6] Halpin K, Young PL, Field HE, Mackenzie JS. Isolation of Hendra virus from pteropid bats: a natural reservoir of Hendra virus. J Gen Virol. 2000;81:1927–32. 10900029 10.1099/0022-1317-81-8-1927

[7] Bowden TR, Westenberg M, Wang L-F, Eaton BT, Boyle DB. Molecular characterization of Menangle virus, a novel paramyxovirus which infects pigs, fruit bats and humans. Virology. 2001;283:358–73. 10.1006/viro.2001.0893 11336561

[8] Chua KB, Koh CL, Hooi PS, Wee KF, Khong JH, Chu BH, Isolation of Nipah virus from Malaysian Island flying-foxes. Microbes Infect. 2002;4:145–51. 10.1016/S1286-4579(01)01522-2 11880045

[9] Chua KB, Wang LF, Lam SK, Eaton BT. Full length genome sequence of Tioman virus, a novel paramyxovirus in the genus *Rubulavirus* isolated from fruit bats in Malaysia. Arch Virol. 2002;147:1323–48. 10.1007/s00705-002-0815-5 12111411

[10] Kim GR, Lee YT, Park CH. A new natural reservoir of hantavirus: isolation of hantaviruses from lung tissue of bats. Arch Virol. 1994;134:85–95. 10.1007/BF01379109 8279962

[11] Bunnell JE, Hice CL, Watts DM, Montrueil V, Tesh RB, Vinetz JM. Detection of pathogenic *Leptospira* spp infections among mammals captured in the Peruvian Amazon basin region. Am J Trop Med Hyg. 2000;63:255–8. 11421373

[12] Arata AA, Vaughn JB, Newell KW, Barth RA, Gracian M. *Salmonella* and *Shigella* infections in bats in selected areas of Colombia. Am J Trop Med Hyg. 1968;17:92–5. 4866292 10.4269/ajtmh.1968.17.92

[13] Heard DJ, Young JL, Goodyear B, Ellis GA. Comparative rectal bacterial flora of four species of flying fox (*Pteropus* sp). J Zoo Wildl Med. 1997;28:471–5. 9523642

[14] Souza V, Rocha M, Valera A, Eguiarte LE. Genetic structure of natural populations of *Escherichia coli* in wild hosts on different continents. Appl Environ Microbiol. 1999;65:3373–85. 10427022 10.1128/aem.65.8.3373-3385.1999 PMC91507

[15] Chua KB. A novel approach for collecting samples from fruit bats for isolation of infectious agents. Microbes Infect. 2003;5:487–90. 10.1016/S1286-4579(03)00067-4 12758277

[16] Heideman PD, Utzurrum RCB. Seasonality and synchrony of reproduction in three species of nectarivorous Philippines bats. Biomedcentral Ecology. 2003. Available from http://www.biomedcentral.com/1472-6785/3/11 10.1186/1472-6785-3-11 PMC305358 14633285

[17] Arguin PM, Murray-Lillibridge K, Mirand MEG, Smith JS, Calaor AB, Rupprecht CE. Serologic evidence of *lyssavirus* infection among bats, the Philippines. Emerg Infect Dis. 2002;8:258–62. 10.3201/eid0803.010330 11927022 PMC2732470

[18] How SJ, Hobson D, Hart CA. Studies in vitro of the nature and synthesis of the cell wall of *Chlamydia trachomatis.* Curr Microbiol. 1984;10:269–74. 10.1007/BF01577140

[19] How SJ, Hobson D, Hart CA, Quayle E. A comparison of the in vitro activity of antimicrobials against *Chlamydia trachomatis* examined by Giemsa and a fluorescent antibody stain. J Antimicrob Chemother. 1985;15:399–404. 10.1093/jac/15.4.399 3891709

[20] Rurangirwa FR, Dilbeck PM, Crawford TB, McGuire TC, McElwain TF. Analysis of the 16S rRNA gene of microorganism WSU8-1044 from an aborted bovine foetus reveals that it is a member of the order *Chlamydiales* proposal of *Waddliaceae* fam. nov., *Waddlia chondrophila* gen. nov., sp. nov. Int J Syst Bacteriol. 1999;49:577–81. 10.1099/00207713-49-2-577 10319478

[21] Everett KDF, Bush RM, Anderson AA. Emended description of the order *Chlamydiales*, proposal of *Parachlamydiaceae* fam. nov. and *Simkaniaceae* fam. nov. each containing one monotypic genus, revised taxonomy of the family *Chlamydiaceae*, including a new genus and five new species, and standards for the identification of organisms. Int J Syst Bacteriol. 1999;49:415–40. 10.1099/00207713-49-2-415 10319462

[22] Griffiths E, Gupta RS. Protein signatures distinctive of chlamydial species: horizontal transfers of cell wall biosynthesis genes *glmU* from archaea to chlamydiae and *murA* between chlamydiae and *Streptomyces.* Microbiology. 2002;148:2541–9. 12177347 10.1099/00221287-148-8-2541

[23] Subtil A, Dautry-Varsat A. Type III secretion system in *Chlamydia* species: identified members and candidates. Microbes Infect. 2000;2:367–9. 10.1016/S1286-4579(00)00335-X 10817638

[24] Thomson JD, Higgins DG, Gibson TJ. CLUSTAL W: improving the sensitivity of progressive multiple sequence alignment through sequence weighting position-specific gap penalties and weight matrix choice. Nucleic Acids Res. 1994;22:4673–80. 10.1093/nar/22.22.4673 7984417 PMC308517

[25] Schachter J, Stephens RS, Timms P, Kuo C, Bavoil PM, Birkelund S, Radical changes to chlamydial taxonomy are not necessary just yet. Int J Syst Evol Microbiol. 2001;51:249. 11211265 10.1099/00207713-51-1-249

[26] Corsaro D, Vallassina M, Venditti D. Increasing diversity within *Chlamydiae.* Crit Rev Microbiol. 2003;29:37–78. 10.1080/713610404 12638718

[27] Corsaro D, Venditti D. Emerging chlamydial infections. Crit Rev Microbiol. 2004;30:75–106. 10.1080/10408410490435106 15239381

[28] Kostanjsek R, Stras J, Drobne D, Avgustin G. *"Candidatus* Rhabdochlamydia porcellionis," an intracellular bacterium from the hepatopancreas of the terrestrial isopod *Porcellio scaber* (Crustacea: Isopoda). Int J Syst Evol Microbiol. 2004;54:543–9. 10.1099/ijs.0.02802-0 15023973

[29] Thao ML, Baumann L, Hess JM, Falk BW, Ng JCK, Gullan PJ, Phylogenetic evidence for two insect-associated chlamydia of the family *Simkaniaceae.* Curr Microbiol. 2003;47:46–50. 10.1007/s00284-002-3953-9 12783192

[30] Fritsche TR, Horn M, Wagner M, Herwig RP, Schleifer K-H, Gautom RK. Phylogenetic diversity among geographically dispersed endosymbionts recovered from clinical and environmental isolates of *Acanthamoeba spp.* Appl Environ Microbiol. 2000;66:2613–9. 10.1128/AEM.66.6.2613-2619.2000 10831445 PMC110588

[31] Horn M, Collingro A, Schmitz-Esser S, Beier CL, Puckhold U, Fartmann B, Illuminating the evolutionary history of chlamydiae. Science. 2004;304:728–30. 10.1126/science.1096330 15073324

[32] Henning K, Schares G, Granzow H, Polster U, Hartmann M, Hotzel H, *Neospora caninum* and *Waddlia chondrophila* strain 2032/99 in a septic stillborn calf. Vet Microbiol. 2002;85:285–92. 10.1016/S0378-1135(01)00510-7 11852195

[33] Bodett TJ, Viggers K, Warren K, Swan R, Conaghty S, Sims C, Wide range of Chlamydiale types detected in native Australian mammals. Vet Microbiol. 2003;96:177–87. 10.1016/S0378-1135(03)00211-6 14519335

[34] Longbottom D, Coulter LJ. Animal chlamydioses and zoonotic implications. J Comp Pathol. 2003;128:217–44. 10.1053/jcpa.2002.0629 12834606

[35] Dilbeck-Robertson P, McAllister MM, Bradway D, Evermann JF. Results of a new serologic test suggest an association of *Waddlia chondrophila* with bovine abortion. J Vet Diagn Invest. 2003;15:568–9. 10.1177/104063870301500609 14667020

[36] Friedman MG, Dvoskin B, Kahane S. Infections with chlamydia-like microorganism *Simkania negevensis*, a possible emerging pathogen. Microbes Infect. 2003;5:1013–9. 10.1016/S1286-4579(03)00188-6 12941393

[37] Birtles RJ, Rowbotham TJ, Storey C, Marrie TJ, Raoult D. Chlamydia-like obligate parasite of free living amoebae. Lancet. 1997;349:925–6. 10.1016/S0140-6736(05)62701-8 9093261

[38] Michel R, Steinert M, Zoller L, Hauroder B, Henning K. Free-living amoebae may serve as hosts for the Chlamydia-like bacterium *Waddlia chondrophila* isolated from an aborted bovine foetus. Acta Protozool. 2004;43:37–42.

[39] Kahane S, Dvoskin B, Mathias M, Friedmann MG. Infection of *Acanthamoeba polyphaga* with *Simkania negevensis* and *S. negevensis* survival within amoebal cysts. Appl Environ Microbiol. 2001;67:4789–95. 10.1128/AEM.67.10.4789-4795.2001 11571186 PMC93233

[40] Essig A, Heinemann M, Simnacher U, Marre R. Infection of *Acanthamoeba castellani* by *Chlamydia pneumoniae.* Appl Environ Microbiol. 1997;63:1396–9. 9097437 10.1128/aem.63.4.1396-1399.1997 PMC168434

